# Low health literacy and psychological symptoms potentially increase the risks of non-suicidal self-injury in Chinese middle school students

**DOI:** 10.1186/s12888-016-1035-y

**Published:** 2016-09-20

**Authors:** Shi-chen Zhang, Fang-biao Tao, Xiao-yan Wu, Shu-man Tao, Jun Fang

**Affiliations:** 1 Department of Maternal and Child Health, School of Public Health, Anhui Medical University, 81th Meishan Road, 230032 Hefei, Anhui Province People’s Republic of China; 2 Department of Toxicology, School of Public Health, Anhui Medical University, 81th Meishan Road, 230032 Hefei, Anhui Province People’s Republic of China; 3 Anhui Provincial Key Laboratory of Population Health & Aristogenics, 81th Meishan Road, 230032 Hefei, Anhui Province People’s Republic of China; 4 Faculty of Pharmaceutical Science, Sojo University, Ikeda 4-22-1, Kumamoto, 860-0082 Japan

**Keywords:** Health literacy, Non-suicidal self-injury, Psychological symptoms, Student

## Abstract

**Background:**

Low health literacy (HL) has been known to be involved in various risk behaviors and mental disorder among adolescent. The purpose of this study was to examine the independent and interactive association between HL and self-reported mental health with non-suicidal self-injury (NSSI) in Chinese middle school students.

**Methods:**

Twenty five thousand three hundred seventy-eight junior and high school students in China were enrolled in this study. The outcomes were self-reported HL, psychological symptoms and NSSI. Logistic regression models were conducted to examine relations between them.

**Results:**

The prevalence of NSSI was 27.5 %. Low HL was significantly associated with NSSI (*OR* = 2.538, 95 % *CI*: 2.335–2.758). Psychological symptoms were significantly positively correlated with NSSI (*OR* = 3.872, 95 % *CI*: 3.637–4.123). Low HL and psychological symptoms were independently and interactively associated with increased risks of NSSI.

**Conclusions:**

These results suggest that Chinese middle school students with low HL and psychological symptoms are intending to exhibit NSSI. The intervention programs of mental health and behavior problems should enhance HL levels and attenuate the severity of psychological symptoms.

**Electronic supplementary material:**

The online version of this article (doi:10.1186/s12888-016-1035-y) contains supplementary material, which is available to authorized users.

## Background

Health literacy (HL) is the degree to which individuals can obtain, process, and understand the basic health information and services they need to make appropriate health decisions [1]. In recent years, HL has gained considerable concern around the world. Ample research support the important role of HL in human health, and it is becoming more and more clear that high HL can improve health conditions and well-being, and reduce poor health outcome. In contrast, low HL competencies will result in less healthy choices, riskier behaviors, poor health conditions and less self-management [2]. In addition, low HL has also been known to be involved in various risk behaviors and mental disorders among adolescent [3, 4].

Non-suicidal self-injury (NSSI) is any intentional, self-directed behavior that causes immediate destruction of body tissues, with no suicidal intent. This behavior is included in a variety of forms, such as cutting, skin carving, burning, severe scratching/abrading, and punching/hitting [5]. A quite high proportion of adolescent who engage in NSSI are supposed to show elevated levels of psychological symptoms, such as depression, anxiety, or major psychiatric disorders [6, 7]. The prevalence of common NSSI ranges from 7 to 37.2 % in students of western countries [8–10], and approximately 17.0 % of students were reported to have NSSI behavior in China [7].

HL is a more useful predictor of an individual’s health status than income, employment status, education level and racial or ethnic group [11, 12]. Many studies have shown that adequate HL, especially mental HL is an important determinant of promoting mental health, particularly for those with major depression, the condition most frequently associated with suicidal behavior [13, 14]. Nevertheless, most of this research is still based on small populations with a focus on the functional HL of patients [15–17]. Also, previously few Chinese studies concerned the relationship between HL and disease outcome, as well as the potential of HL for the prediction of mental health and behavior problems.

The aim of this study was to examine (i) associations of HL and psychological symptoms with NSSI; (ii) the interactive effects of HL and psychological symptoms on NSSI in Chinese middle school students.

## Methods

### Study participants

Convenient cluster sampling was conducted from junior and senior high schools located in 6 cities in China, including both urban and rural regions, as follows: Shenyang (Liaoning Province), Xinxiang (He’nan Province), Guangzhou (South central Guangdong Province), Chongqing (one of China’s four direct-controlled municipalities), Xinjiang (Xinjiang Uyghur Autonomous Region) and Yangjiang (Southwest of Guangdong Province). Eight schools in each city were selected including two junior schools and two senior schools from rural regions and two junior schools and two senior schools from urban regions. This study was conducted from December 2013 to May 2014.

A total of 27044 students (Aged 11–19 years) were recruited in this study, participants were from grade 7–12 in junior and senior high schools. In the participating schools, 774 of the 27044 sampled students were excluded from the study because of their absence from school on the day of the survey or reluctance to respond to the questionnaires. We finally received 26152 (96.7 %) questionnaires, in which 25378 (97.0 %) were valid (missing data > 5 % was discard). In addition, the participants of six cities were 3389 (Shenyang), 3375 (Xinxiang), 2904 (Guangzhou), 6394 (Chongqing), 3179 (Xinjiang), and 6137 (Yangjiang), respectively.

### Ethics Statement

Approval for the study was granted by the Ethics Committee of Anhui Medical University (Mar. 1, 2014; approval number 20140087). Permission for the study was requested from schools, parents, and students before completion of the surveys. During the organization period, we had signed informed consent with each participating school. The students were allowed to participate in the study upon receiving completed consent form from their parents. One week prior to screening day, the parents were informed of the study through a notice sent home from the schools asking them to contact the teachers by phone if they wish their child to participate in the survey. Prior to the formal investigation, the team members introduced the aims and procedures of the study to the students, and provided an opportunity for them to ask questions. If they were not willing to participate, they were allowed to withdraw from the study. Consent to participate in the study was recorded in a separate consent form with the questionnaire, and it was confirmed upon completion and return of the questionnaire.

### Design of questionnaires

A self-report questionnaire was used to collect information on socio-demographic variables, HL, psychological symptoms, and NSSI during a 20–30 min session in the classroom. The following socio-demographic characteristics were obtained: age, gender, grade (junior or senior high schools), residential background (urban or rural area), household structure (only-child or more than one child), parents’ educational level, and self-reported family economy.

HL was assessed with a reliable questionnaire: the Chinese Adolescent Interactive Health Literacy Questionnaire (CAIHLQ), and the reliability and validity of CAIHLQ have been demonstrated in previous study [18]. It consists of 31 questions grouped into 6 domains, including 6 questions assessing physical activities (e.g. ‘Follow a planned exercise program.’), 5 for interpersonal relationship (e.g. ‘Take times with your family or friends.’), 6 for stress management (e.g. ‘Balance time between study and play.’), 4 for self- actualization (e.g. ‘Feel each day is very meaningful.’), 5 for health awareness (e.g. ‘Containing sugars and food continuing sugar.’), and 5 for dietary behavior (e.g. ‘Eat 200–400 g of fresh fruit each day.’). All of these questions have five answer categories (never and no desire, never but with desire, occasionally and irregularly, often, and routinely), and the total score is standard converted to a score that ranges from 31 to 155, with lower scores indicating inadequate HL (see Additional file 1). Variance cumulative contribution rate was 63.847 %; internal consistency test showed that the total questionnaire Cronbach’s α was 0.937, Cronbach’s α of each dimension was 0.752 to 0.898; correlation analysis showed that Pearson correlation coefficients of each item and the total score were all above 0.4, and the Pearson correlation coefficient of each item and its dimension were more than 0.5, all the indicators were at the significant level and had a good reliability and validity. Students in this study were categorized as low, medium and high HL when their scores were < *P*_*25*,_*P*_*25*_- *P*_*75*_ and > *P*_*75*_ respectively.

Psychological symptoms were measured using the psychological domain of the Multidimensional Sub-health Questionnaire of Adolescents (MSQA) [19], which consists of 39 items in 3 subscales: 17 questions for emotional symptoms (e.g. ‘Do you always feel nervous?’); 9 for conduct symptoms (e.g. ‘Do you always have the impulse to damage something?’); and 13 for social adaptation symptoms (e.g. ‘Could you always not be suited for school life?’). Each participant was asked to rate each item according to a 6-point scale where ≥ 3 months, ≥ 2 months, ≥ 1 month, ≥ 2 weeks, ≥ 1 weeks, and none or < 1 week. In calculation step, the symptom duration time lasting ≥ 1 month, lasting ≥ 2 months, or lasting ≥ 3 months was assigned renewedly to 1 and the others to 0. In accordance with the national norm established for MSQA of adolescents in China [20], the 90th percentile of national norm in total score and six subscales scores were selected as the cut-off points, which was eight for psychological problems in this study. Therefore, the number of score ‘1’ ≥ 8 was supposed to have psychological symptoms. The MSQA has been reported by various groups to be a valid and reliable method to explore determinants and the current state of adolescents’ psychological health status [21–24].

NSSI was elicited in a self-report by answering: Have you ever harmed yourself in a way that was deliberate, but not intended as a means to take your life in the past 12 months? The response options were “yes or no” [8, 25]. Eight NSSI behaviors were presented, and the details of the questions were as follows: (1) Have you ever hit yourself?; (2) Have you ever pulled your hair yourself?; (3) Have you ever banged your head or fist against something?; (4) Have you ever pinched or scratched yourself?; (5) Have you ever bitten yourself?; (6) Have you ever cut or pierced yourself?; (7) Have you ever exposed yourself to smoke, fire, and flames or come in contact with heat and hot substances?; and (8) Have you ever ingested a toxic substance or object? All students who answered yes (one or more times) were judged as having NSSI behaviors. In the previous study, Cronbach’s alpha coefficient for the NSSI was reported to be 0.776 [7].

### Statistical analysis

Statistical analysis was performed using SPSS ver. 13.0 for Windows (SPSS, Inc., Chicago, IL). Cronbach’s alpha analysis was performed to determine the reliability of the survey. The Chi-square analysis of variance was used to assess group differences with respect to their statistical significance, and effect sizes were calculated. The logistic regression analysis was performed to examine the independent and interactive relationships between HL, psychological symptoms, and NSSI. Analyses were adjusted to control for key demographic and socioeconomic variables (i.e., gender, grade, registered residence, household structure, parents’ educational level, and self-reported family economy). Statistical significance was set at *P* < 0.05.

## Results

### Reliability analysis

Cronbach’s alpha coefficient of internal consistency for the present study was shown to have a high value, with the alpha coefficients of 0.934 for overall CAIHLQ and 0.745–0.884 for six subscales, and 0.807 for NSSI, which is similar to previous researches [7, 18].

### Univariate analysis

Participants had a mean age of 15.18 years (*SD* = 1.79), and the overall CAIHLQ mean score for all participants was 103.55 ± 23.91, and the value of *P*_*25*_ and *P*_*75*_ were 89, 119, respectively. The scores of CAIHLQ were normally distributed and the variability in the data was homogeneous. Table 1 presents the prevalence of NSSI by frequency characteristics. A total of 6971 (27.5 %) students reported NSSI behaviors in the past 12 months. The total rate of NSSI revealed statistically significant differences by gender [male (28.6 %) vs. female (26.4 %), *P* < 0.001], and the same tendency was shown on grade, registered residence, household structure, mother’s educational level and perceived family economical status (*P* < 0.05 for each), while NSSI revealed no statistically significant differences by father’s educational level (*P* > 0.05, Table 1). In addition, there was a marked difference between HL [high HL (17.3 %) vs. medium HL (28.6 %) vs. low HL (34.7 %), *V* = 0.140] and psychological symptoms [no (21.0 %) vs. yes (50.7 %), *φ* = 0.275] with NSSI (*P* < 0.01for each, Table 1).

**Table 1:** Frequency characteristics of the sample in the current study

Variable	Total Sample (n = 25378)	NSSI		*x* ^*2*^	*φ*/*V*
		No (n = 18407)	Yes (n = 6971)		
Gender				14.534 ﻿^*﻿*﻿﻿*^	-0.024 ^***^
Male	12325 (48.6)	8804 (71.4)	3521 (28.6)		
Female	13053 (51.4)	9603 (73.6)	3450 (26.4)		
Grade				4.565 ^*^	−0.013 ^*^
Junior school	13179 (51.9)	9483 (72.0)	3696 (28.0)		
Senior high school	12199 (48.1)	8924 (73.2)	3275 (26.8)		
Registered residence				5.836 ^*^	−0.015 ^*^
Rural	11484 (45.3)	8244 (71.8)	3240 (28.2)		
Urban	13894 (54.7)	10163 (73.1)	3731 (26.9)		
Household structure				4.151 ^*^	0.013 ^*^
Only child	12514 (49.3)	9149 (73.1)	3365 (26.9)		
More than one child	12864 (50.7)	9258 (72.0)	3606 (28.0)		
Father’s educational level ^a1^				2.969	−0.011
< High school degree	12781 (50.4)	9221 (72.1)	3560 (27.9)		
≥ High school degree	11800 (46.5)	8629 (73.1)	3171 (26.9)		
Mother’s educational level ^a2^				7.413 ^**^	−0.017 ^**^
< High school degree	14261 (56.2)	10261 (72.0)	4000 (28.0)		
≥ High school degree	10466 (41.2)	7694 (73.5)	2772 (26.5)		
Self-reported family economy				49.988 ^***^	0.044 ^***^
Bad	3732 (14.7)	2530 (67.8)	1202 (32.2)		
General	17064 (67.2)	12538 (73.5)	4526 (26.5)		
Good	4582 (18.1)	3339 (72.9)	1243 (27.1)		
HL				499.625 ^***^	0.140 ^***^
High	6159 (24.3)	5091 (82.7)	1068 (17.3)		
Medium	12674 (49.9)	9045 (71.4)	3629 (28.6)		
Low	6545 (25.8)	4271 (65.3)	2274 (34.7)		
Psychological symptoms				1923.561 ^***^	0.275 ^***^
No	19813 (78.1)	15661 (79.0)	4152 (21.0)		
Yes	5565 (21.9)	2746 (49.3)	2819 (50.7)		

Statistical methods: Chi-square test, *φ*/*V* is effect sizes. HL is health literacy; NSSI is non-suicidal self- injury

^***^ *P* < 0.001

^**^ *P* < 0.01

^*^ *P* < 0.05

^a1^ 797 students have no father

^a2^ 651 students have no mother

As shown in Table 2, the prevalence rates of eight sorts of NSSI were presented for gender. A total of 4386 (17.3 %) students reported that they ever had banged their head or fist against something in the past 12 months. The most reported behaviors were banging head, hitting, and scratching. In terms of gender differences in type of NSSI endorsed, females were more likely to engage in scratching (*x*^*2*^ = 189.405), biting (*x*^*2*^ = 84.766) and cutting (*x*^*2*^ = 59.657), but males were more likely to engage in pulling hair (*x*^*2*^ = 29.842), banging head (*x*^*2*^ = 337.334), burning (*x*^*2*^ = 40.719) and poisoning (*x*^*2*^ = 25.572) (*P* < 0.01for each). No significant differences were found in hitting (Q1) between male and female (*P* > 0.05, Table 2).

**Table 2:** Proportion of NSSI behaviors in adolescents by gender

Methods	Total Sample	Male	Female	*x* ^*2*^	*φ*
1. Have you ever hit yourself?				0.012	0.001
No	21964 (86.5)	10670 (48.6)	11294 (51.4)		
Yes	3414 (13.5)	1655 (48.5)	1759 (51.5)		
2. Have you ever pulled your hair yourself?				29.842 ^***^	−0.034 ^***^
No	22962 (90.5)	11024 (48.0)	11938 (52.0)		
Yes	2416 (9.5)	1301 (53.8)	1115 (46.2)		
3. Have you ever banged your head or fist against something?				337.334 ^***^	−0.115 ^***^
No	20992 (82.7)	9642 (45.9)	11350 (54.1)		
Yes	4386 (17.3)	2683 (61.2)	1703 (38.8)		
4. Have you ever pinched or scratched yourself?				189.405^***^	0.086 ^***^
No	22779 (89.8)	11395 (50.0)	11384 (50.0)		
Yes	2599 (10.2)	930 (35.8)	1669 (64.2)		
5. Have you ever bitten yourself?				84.766^***^	0.058 ^***^
No	23688 (93.3)	11687 (49.3)	12001 (50.7)		
Yes	1690 (6.7)	638 (37.8)	1052 (62.2)		
6. Have you ever cut or pierced yourself?				59.657^***^	0.048 ^***^
No	24034 (94.7)	11810 (49.1)	12224 (50.9)		
Yes	1344 (5.3)	515 (38.3)	829 (61.7)		
7. Have you ever exposed yourself to smoke, fire, and flames or come in contact with heat and hot substances?				40.719^***^	−0.040 ^***^
No	24560 (96.8)	11838 (48.2)	12722 (51.8)		
Yes	818 (3.2)	487 (59.5)	331 (40.5)		
8. Have you ever ingested a toxic substance or object?				25.572^***^	−0.032 ^***^
No	25017 (98.6)	12102 (48.4)	12915 (51.6)		
Yes	361 (1.4)	223 (61.8)	138 (38.2)		

Statistical methods: Chi-square test

^***^ *P* < 0.001

Table 3 presents the scores of the HL by frequency characteristics. The junior students had significantly higher HL scores than senior high school students, and the same tendency was shown on urban residence and only child family (*P* < 0.001for each). In addition, significant associations of parental education and perceived family economical status with HL were observed; respondents whose parents had a higher education level showed higher HL scores, and students with higher family income had higher HL level (*P* < 0.001for each). The prevalence of psychological symptoms was 21.9 %, and the effect size of psychological symptoms and HL was 0.261 (*P* < 0.001, Table 3).

**Table 3:** Description of students with health literacy according to CAIHLQ

Variable	Total Sample (*n* = 25378)	Low HL (*n* = 6545)	Medium HL (*n* = 12674)	High HL (*n* = 6159)	*x* ^*2*^	*V*
Gender					156.118 ^***^	0.078 ^***^
Male	12325 (48.6)	3259 (26.4)	5706 (46.3)	3360 (27.3)		
Female	13053 (51.4)	3286 (25.2)	6968 (53.4)	2799 (21.4)		
Grade					337.038 ^***^	0.115 ^***^
Junior school	13179 (51.9)	2868 (21.8)	6610 (50.2)	3701 (28.1)		
Senior high school	12199 (48.1)	3677 (30.1)	6064 (49.7)	2458 (20.1)		
Registered residence					306.126 ^***^	0.110 ^***^
Rural	11484 (45.3)	3462 (30.1)	5716 (49.8)	2306 (20.1)		
Urban	13894 (54.7)	3083 (22.2)	6958 (50.1)	3853 (27.7)		
Household structure					174.235 ^***^	0.083 ^***^
Only child	12514 (49.3)	2927 (23.4)	6130 (49.0)	3457 (27.6)		
More than one child	12864 (50.7)	3618 (28.1)	6544 (50.9)	2702 (21.0)		
Father’s educational level ^a1^					352.694 ^***^	0.118 ^***^
< High school degree	12781 (50.4)	3783 (29.6)	6405 (50.1)	2593 (20.3)		
≥ High school degree	11800 (46.5)	2514 (21.3)	5906 (50.1)	3380 (28.6)		
Mother’s educational level ^a2^					553.304 ^***^	0.144 ^***^
< High school degree	14261 (56.2)	4198 (29.4)	7258 (50.9)	2805 (19.7)		
≥ High school degree	10466 (41.2)	2106 (20.1)	5148 (49.2)	3212 (30.7)		
Self-reported family economy					630.169 ^***^	0.158 ^***^
Bad	3732 (14.7)	1413 (37.9)	1659 (44.5)	660 (17.7)		
General	17064 (67.2)	4281 (25.1)	8867 (52.0)	3916 (22.9)		
Good	4582 (18.1)	851 (18.6)	2148 (46.9)	1583 (22.9)		
Psychological symptoms					1726.544 ^***^	0.261 ^***^
No	19813 (78.1)	4049 (20.4)	10094 (50.9)	5670 (28.6)		
Yes	5565 (21.9)	2496 (44.9)	2580 (46.4)	489 (8.8)		

Statistical methods: Chi-square test. HL is health literacy

^***^ *P* < 0.001

^a1^ 797 students have no father

^a2^ 651 students have no mother

### Multiple logistic regression analysis

Results from multiple logistic regression analysis indicated that, low HL was significantly associated with NSSI (*OR* = 2.538, 95%*CI*: 2.335–2.758), and psychological symptoms were significantly positively correlated with NSSI (*OR* = 3.872, 95%*CI*: 3.637–4.123), which also indicated a dose-response relationship (*P* < 0.001 for each, Table 4). Further, these associations were also seen after adjusting for gender, grade, registered residence, household structure, perceived family economical, and parents’ educational level (Table 4).

**Table 4:** Associations of psychological symptoms, HL, and NSSI among junior and high school students

Variables	NSSI		
	*n* (%)	Crude OR (95 % CI)	Adjusted OR (95 % CI) ^a^
Psychological symptoms			
No	4152 (21.0)	Ref.	Ref.
Yes	2819 (50.7)	3.872 (3.637–4.123) ^***^	4.019 (3.769–4.285) ^***^
HL			
High	1068 (17.3)	Ref.	Ref.
Medium	3629 (28.6)	1.913 (1.772–2.064) ^***^	1.939 (1.794–2.094) ^***^
Low	2274 (34.7)	2.538 (2.335–2.758) ^***^	2.555 (2.345–2.785) ^***^

HL is health literacy; NSSI is non-suicidal self- injury

*OR* odds ratio; *CI* confidence interval

^***^ *P* < 0.001 compared with referent

^a^ Adjusted for gender, grade, registered residence, household structure, parents’ educational level, and perceived family economical

As shown in Table 5, the interaction effects of HL and psychological symptoms with NSSI, and the crude and adjusted *OR* (95%*CI*), were presented for each group in comparison with the reference group (no psychological symptoms and high HL) for NSSI, respectively. A significant additive interaction was observed between HL and psychological symptoms (*P* < 0.001). In particular, the students with low HL and psychological symptoms had the highest risks of NSSI (*OR* = 6.036, 95%*CI*: 5.788–7.719), which also indicated a dose-response relationship (*P* < 0.001 for each). Further, these associations were also seen in the adjusted models (Table 5).

**Table 5:** Odds ratio (95 % *CI*) associated with the interaction of HL and psychological symptoms on NSSI among junior and high school students

Psychological symptoms	HL	NSSI			*P* ^*^
		*n* (%)	Crude OR (95 % CI)	Adjusted OR ^a^ (95 % CI)	
No	High	861 (15.2)	Ref.	Ref.	<0.001
	Medium	2289 (22.7)	1.638 (1.503–1.756) ^***^	1.676 (1.536–1.828) ^***^	
	Low	1002 (24.7)	1.837 (1.659–2.033) ^***^	1.877 (1.692–2.082) ^***^	
Yes	High	207 (42.3)	4.100 (3.379–4.975) ^***^	4.264 (3.511–5.180) ^***^	
	Medium	1340 (51.9)	5.804 (5.216–6.459) ^***^	6.242 (5.588–6.972) ^***^	
	Low	1272 (51.0)	6.036 (5.429–6.710) ^***^	6.446 (5.788–7.719) ^***^	

HL is health literacy; NSSI is non-suicidal self-injury

^*^ *P*-value of interaction between HL and psychological symptoms on NSSI in multiplicative model

^***^ *P* < 0.001 compared with referent

^a^ Adjusted for gender, grade, registered residence, household structure, parents’ educational level, and perceived family economy

## Discussion

The findings of the present study suggest that HL and psychological symptoms are independently and interactively associated with NSSI. Namely, low HL and psychological symptoms are correlated with an increased prevalence of NSSI.

NSSI among adolescents is common and the rate may be increasing [26]. Recent studies have found that 51 and 55.6 % of students in US and Spain respectively, have engaged in some type of NSSI [27, 28]. In the current study, the prevalence of 27.5 % of NSSI is lower than previous study in 4405 Chinese students (29.2 %) [29], and similar to that reported in 12,068 adolescents of 11 European countries (27.6 %) [30]. Our results indicated that junior students had significantly higher prevalence of NSSI than senior high school students, as reported in previous studies, namely NSSI seems to occur during early adolescence [31]. Steinberg et al. suggested that the “impulsive choice” is greatest during early - to mid - adolescence [32], which may be associated with the occurrence of NSSI during early adolescence. Also, these behavioral changes probably reflect the substantial development of brain networks—including structures in the prefrontal cortex and other cortical and subcortical regions, along with their interaction, leads to behavior that is biased towards risk and emotional reactivity during the adolescent period [33].

Importantly, our study found gender differences in the prevalence of NSSI among adolescents. Previous studies reported that female adolescents showed higher frequencies of NSSI than male adolescents [27, 29]. However, our findings indicated higher frequency of NSSI in male adolescents. So the gender differences of NSSI are not yet clear, which need further investigations to be elucidated. Regarding the specific NSSI behaviors, female showed higher frequency of self-cutting and skin damage by using methods of such as scratching and biting, compared to male. In contrast, there was a gender-specific over-representation of self-burning and self-hitting in male adolescents. These findings are consistent with the previous study [30].

Demographic features such as age, gender, educational level, ethnicity, family income and geographic location, etc, have been found to relate to HL [34–36]. In this study, students with rural residence with lower family income and those of non-only children had lower scores of HL, and males had significantly higher HL scores than females, meanwhile, junior students had higher levels of HL than senior high school students (*P* < 0.001 for each), these findings are congruent with results from the 2008 HL Survey of Chinese residents [37]. Increasing evidence is suggesting that low HL is associated with numerous adverse health outcomes [38–41]. Our results suggest that inadequate HL was associated with significantly increasing psychological symptoms, and the prevalence of NSSI. Moreover, low HL and psychological symptoms increase the risk of NSSI independently and also synergistically in Chinese middle school students.

The current study was a representative nationwide epidemiologic study with large samples; however, there were some limitations should be considered. First, the reliance on self-report nature of the data, and recall and reporting biases could not be avoided. Moreover, because this study used a cross-sectional design, the results do not imply causality but suggest the possible association of HL and psychological symptoms with students’ NSSI. Longitudinal studies may thus need to clarity causal relationship of HL and mental health with NSSI in the future study.

## Conclusions

Taken together, the present study highlights the importance of associations of HL and psychological symptoms with NSSI in Chinese middle school students. Our results indicate that low HL is significantly positively correlated with mental behavior problems. Furthermore, low HL and psychological symptoms are associated with increased risks of NSSI. Further research is needed to clarify the potential mechanisms underlying this relationship. These findings may suggest that intervention program for students should focus on enhancing HL and attenuating the severity of psychological symptoms to maintain their desirable healthy status and well-being.

## Additional file

Additional file 1: English version and Chinese version of CAIHLQ. (DOC 569 kb)

## Abbreviations

CAIHLQ: Chinese Adolescent Interactive Health Literacy Questionnaire
CI: confidence interval
HL: health literacy
MSQA: Multidimensional Sub-health Questionnaire of Adolescents
NSSI: non-suicidal self-injury
OR: odds ratio
