# Supplementary material for: Low health literacy and psychological symptoms potentially increase the risks of non-suicidal self-injury in Chinese middle school students
Source: BMC Psychiatry. 2016 Sep 20;16:327. doi: 10.1186/s12888-016-1035-y (PMC5028961; doi:10.1186/s12888-016-1035-y)
Supplement: Additional file 1: — English version and Chinese version of CAIHLQ. (DOC 569 kb) [file 12888_2016_1035_MOESM1_ESM.doc]

Chinese Adolescent Interactive Health Literacy Questionnaire (CAIHLQ)
(Please select only one answer.)
Have you had done these things?	Never and no desire	Never but with desire	Occasionally and irregularly	Often	Routinely	
1. Follow a planned exercise program.						
2.	Exercise vigorous intensity for accumulate at least 60 minutes physical activity daily.						
3. Exercise moderate intensity for accumulate at least 60 minutes physical activity daily.						
4. Check own pulse rate after exercising immediately.						
5. Get exercise during usual daily activities (such as using stairs instead of elevators, by bicycle instead of by bus)						
						
6. Take part in leisure-time (recreational) physical activities (such as yoga, swimming, dancing).						
7. Get along well with your family.						
8. Maintain meaningful and fulfilling relationships with fiends or classmates.						
9. Show concern, love and warmth to others (such as friends, classmates, strangers). 						
10. Get support from a network of caring people.						
						
11. Take times with your family or friends.						
12. Practice relaxation or meditation for 15-20 minutes daily.						
13. Get enough sleep.						
14. Concentrate on pleasant thoughts at bedtime.						
15. Use specific methods to control your stress (such as music, sports, and pour forth your feeling to someone).						
						
16. Balance time between study and play.						
17. Pace myself to prevent tiredness.						
18. Have courage to challenge new things.						
19. Work toward long-term goals in your life.						
20. Look forward to the future.						
						
21. Feel each day is very meaningful.						
22. Read or watch TV programs about improving health.						
23. Don't drink soft drinks.						
24. Read labels to identify nutrients, fats, and sodium content in packaged food.						
25. Containing sugars and food continuing sugar.						
						
26. Eat western fast food no more than twice a month.						
27. Eat 80-100 gram of meet each day (equal to the size of adult's palm )						
28. Eat 200-400 gram of fresh fruit each day (equal to the size of adult's 2-3 fist).						
29. Eat 200-400 gram of vegetables each day (equal to the size of adult's 2-3 fist).						
30. Eat 250-400 gram of cereal each day (equal to 3-4 small bowls of rice/noodle).						
31. Eat 2-3 servings of dairy products each day (equal to 250 ml of milk or 2 pieces of cheese).						


ÖÐ¹úÇàÉÙÄê»¥¶¯ÐÔ½¡¿µËØÑøÆÀ¶¨Á¿±í
µ¥Ñ¡Ìâ£¬Ã¿ÌâÇëÑ¡ÔñÒ»¸ö´ð°¸¡£
Äã×ö¹ýÒÔÏÂÕâÐ©ÊÂÇéÂð£¿	Ã»×ö¹ýÒ²Ã»Ïë×ö	Ã»×ö¹ýµ«Ïë×ö	×ö¹ýµ«Ã»ÓÐ¹æÂÉ	¾­³£ÕâÑù×ö	Ò»Ö±¶¼ÕâÑù×ö	
1. ÓÐ¼Æ»®µÄ½øÐÐÌåÓý¶ÍÁ¶						
2. Ã¿Ìì»á×ö60·ÖÖÓ£¨Ê±¼ä¿ÉÀÛ¼Æ£©¸ßµÈÇ¿¶ÈµÄÔË¶¯£¨Èç´òÀºÇò¡¢ÌøÉþ¡¢Ìß×ãÇò¡¢ÈüÅÜµÈ£©						
3. Ã¿Ìì»á×ö60·ÖÖÓ£¨Ê±¼ä¿ÉÀÛ¼Æ£©ÖÐµÈÇ¿¶ÈµÄÔË¶¯£¨ÈçÖÐËÙÅÜ¡¢¿ì×ß¡¢Æ¹ÅÒÇòµÈ£©						
4. ÔË¶¯ºó»áÁ¢¼´×Ô²âÂö²«						
5. ÄÜ¹»½«ÔË¶¯ÈÚÈëÓÚÈÕ³£Éú»îÖÐ£¨Èç×ßÂ¥ÌÝ´úÌæµçÌÝ¡¢Æï×ÔÐÐ³µ´úÌæ×ø³µ£©						
						
6. ²Î¼ÓÓéÀÖÀàµÄÌåÓýÏîÄ¿£¨Èç½¡ÃÀ²Ù¡¢Îèµ¸¡¢è¤Ù¤µÈ£©						
7. ÄÜÓë¼ÒÈËºÍÄÀÏà´¦						
8. ÄÜÓëÅóÓÑ»òÍ¬Ñ§±£³ÖÁ¼ºÃµÄÈË¼Ê¹ØÏµ						
9. ¾­³£¹ØÐÄ°®»¤¡¢ÎÂÅ¯ËûÈË£¨ÈçÍ¬Ñ§¡¢ÅóÓÑ¡¢Ä°ÉúÈËµÈ£©						
10. µ±ÄãÓöµ½À§ÄÑÓÇÂÇµÄÊÂÇéÊ±£¬ÄÜµÃµ½ËûÈËµÄ°ïÖú						
						
11. »á³é³öÊ±¼äÓë¼ÒÈË»òÅóÓÑÔÚÒ»Æð						
12. Ã¿Ìì»á³é³ö15¡«20·ÖÖÓÈ¥·ÅËÉÐÄÇé¡¢È¥É¢ÐÄ»òÚ¤Ïë						
13. ÄÜ±£Ö¤³ä×ãµÄ£¨×ÔÎÒ¸Ð¾õ£©Ë¯Ãß						
14. Ã¿ÌìÈëË¯Ç°ÏëÐ©Óä¿ìµÄÊÂÇé						
15. Æ½Ê±»á×öÒ»Ð©Ïû³ýÑ¹Á¦µÄÊÂÇé£¨ÈçÔË¶¯¡¢ÇãËß¡¢ÌýÒôÀÖµÈ£©						
						
16. ÄÜ¹»Æ½ºâÑ§Ï°ºÍÒµÓàÓéÀÖµÄÊ±¼ä						
17. Éú»îÖÐÄã»á×¢Òâµ÷½Ú×Ô¼º£¬·ÀÖ¹Æ£ÀÍ						
18. ¶ÔÐÂµÄÊÂÎïÓÂÓÚÌôÕ½						
19. Å¬Á¦Ïò×Å×Ô¼ºµÄÈËÉúÄ¿±êÇ°½ø						
20. ¶Ô×Ô¼ºµÄÎ´À´³äÂúÏ£Íû						
						
21. Ã¿Ò»Ìì¶¼¸Ð¾õ¹ýµÃºÜÓÐÒâÒå						
22. ¾­³£¿´¹ØÓÚÌá¸ß½¡¿µµÄÐû´«ºÍµçÊÓ½ÚÄ¿						
23. ÄÜ×öµ½²»ºÈÈíÒûÁÏ£¨ÈçÌ¼ËáÒûÁÏ¡¢ÔË¶¯ÒûÁÏ¡¢¹ûÖ­ÒûÁÏµÈ£©						
24. Âò°ü×°Ê³Æ·Ê±£¬×¢Òâ¿´°ü×°´üºóµÄÓªÑø³É·Ö±í£¬È·ÈÏÖ¬·¾¡¢ÑÎ·ÖµÈº¬Á¿						
25. ¿ØÖÆÉãÈ¡ÌÇ·Ö¹ý¶àµÄÊ³Îï£¨ÈçÄÌÓÍµ°¸â¡¢Åò»¯Ê³Æ·¡¢ÒûÁÏµÈ£©						
						
26. Ã¿ÔÂ³ÔÎ÷Ê½¿ì²Í£¨Èçºº±¤¡¢ÊíÌõ¡¢Õ¨¼¦µÈ£©²»³¬¹ý2´Î						
27. Ã¿Ìì¶¼³Ô80¡«110¿ËµÄÈâÀà£¨Ïàµ±ÓÚ1¸öÆÕÍ¨³ÉÄêÈËµÄÊÖÕÆÐÄµÄ´óÐ¡¼°ºñ¶È£©						
28. Ã¿Ìì¶¼³Ô200¡«400¿ËÐÂÏÊË®¹û£¨Ïàµ±2¡«3¸öÆÕÍ¨³ÉÄêÈËÈ­Í·´óÐ¡£©						
29. Ã¿Ìì¶¼³Ô200¡«400¿ËÊß²Ë£¨Ïàµ±2¡«3¸öÆÕÍ¨³ÉÄêÈËÈ­Í·´óÐ¡£©						
30. Ã¿Ìì¹ÈÀà£¨ÈçÃ×·¹¡¢ÃæÊ³µÈ£©¶¼ÄÜ³Ô250¡«400¿Ë£¨Ïàµ±ÓÚ3¡«4Ð¡ÍëÃ×·¹/ÃæÌõ»ò3¡«4¸öÂøÍ·£©						
31. Ã¿Ìì¶¼ºÈ2¡«3·ÝÈéÖÆÆ·£¨ÈçÅ£ÄÌ¡¢ËáÄÌ¡¢ÄÌÀÒµÈ£©£¨Ïàµ±ÓÚ250ºÁÉýÅ£ÄÌ/ËáÄÌ»ò2Æ¬ÄÌÀÒ£©						
